# Hospital Pharmacists and Olive Oil: Official Tests

**Published:** 1914-12-05

**Authors:** 


### Hospital Pharmacists and Olive Oil: Official Tests.

An evening meeting of the Public Pharmacists' and Dispensers' Association was held at St. Bride Institute, London, last week, on November 26. Mr. J. Hassall France was in the chair. Others present included Messrs. Kinsman, Lindsey, Lindsay, Howell, Thompson, Jenkin, and G. W. Gibson, hon. sec.

Mr. C. Edward Sage, F.I.C., Ph.C., delivered a lecture on "Olive Oil," illustrated with lantern slides. The cultivation of olives was first dealt with, and then methods of obtaining the oil from the fruits, purification of the oil, and finally the tests by which genuine olive oil may be distinguished from adulterated oils. Our chief sources of supply are Italy, the South of France, Spain, and Algeria. The olives are usually hand picked, and crushed in hydraulic presses after first being packed in bags—the expressed oil is afterwards purified by various methods.

The lecturer advised his audience to try to pay a visit to the London Docks, where the variety and immensity of our imports of olive oil and other articles would astound a stranger to them. Mention was made of the tests, official in the new Pharmacopœia, for distinguishing pure olive oil from adulterated.

The refractometer is likely to be used only by works dealing with huge quantities, but for the comparatively small quantity used for institutions the best tests were perhaps to estimate the iodine number, and also to try whether or not the oil made a satisfactory preparation using the formula for ung. hydrarg. nitrat. B.P., and lead plaster. Precautions should be taken to store olive oil in a cool and dark place away from air, which tends to lighten the acidity (oleic acid).

Adulterants of olive oil are chiefly arachis, sesame, and cottonseed oil; and those hospital pharmacists who were able to attend the lecture should be in a better position to determine the genuineness or otherwise of any product submitted to them by contractors as olive oil. A good discussion ensued, and the lecturer replied fully to the many questions which were raised by his audience.

